# A Sensitivity Analysis of fMRI Balloon Model

**DOI:** 10.1155/2015/425475

**Published:** 2015-05-11

**Authors:** Chadia Zayane, Taous Meriem Laleg-Kirati

**Affiliations:** Computer, Electrical and Mathematical Sciences and Engineering, King Abdullah University of Science and Technology (KAUST), Thuwal 23955-6900, Saudi Arabia

## Abstract

Functional magnetic resonance imaging (fMRI) allows the mapping of the brain activation through measurements of the Blood Oxygenation Level Dependent (BOLD) contrast. The characterization of the pathway from the input stimulus to the output BOLD signal requires the selection of an adequate hemodynamic model and the satisfaction of some specific conditions while conducting the experiment and calibrating the model. This paper, focuses on the identifiability of the Balloon hemodynamic model. By identifiability, we mean the ability to estimate accurately the model parameters given the input and the output measurement. Previous studies of the Balloon model have somehow added knowledge either by choosing prior distributions for the parameters, freezing some of them, or looking for the solution as a projection on a natural basis of some vector space. In these studies, the identification was generally assessed using event-related paradigms. This paper justifies the reasons behind the need of adding knowledge, choosing certain paradigms, and completing the few existing identifiability studies through a global sensitivity analysis of the Balloon model in the case of blocked design experiment.

## 1. Introduction

Functional Magnetic Resonance Imaging (fMRI) technique has been the area of great interest since it measures the activity of the brain, which gives much insight on brain functions. The contrast agent in fMRI is based on the rate of oxygen consumption in the brain through the Blood Oxygenation Level Dependent (BOLD) signal. Whether the aim is to characterize a region of the brain or to compare different regions and/or subjects, it is necessary to link the BOLD indirect measure to the stimulus.

Several studies have considered modeling the hemodynamic response of the brain. These models fall in two categories: general linear modeling-based detection methods, which assume a known shape of the hemodynamic response function (HRF) and estimation ones which track the most appropriate HRF form in regions eliciting evoked activity in response to stimulus presentation. The first class of models uses statistical tools (discrepancy) to fit some basis functions (Poisson function [1], Gaussian function [2], Gamma function [3], inverse Logit function [4],…) to the BOLD for the aim of activation detection. The second is more concerned with the physiological aspects that underlie the BOLD transients. It is natural at this point to think that understanding the brain functions requires a much more elaborated framework to map the neural activity to changes in the measured BOLD signal than a convolution by some predefined hemodynamic response functions. Furthermore, it has been shown [5–7] that to be more consistent, this mapping has to encompass nonlinear effects to describe more accurately the pathway from neural activation to the BOLD.

Among the nonlinear hemodynamic models, linking changes in the physiological variables, namely, the blood flow, the volume, and the oxygenation level to the neural activity in a localized area of the brain, is the Balloon model. This model has been first proposed by Buxton et al. [6]. Several variants of the Balloon model have been developed such as Friston et al.'s [8] variant considered in this paper. The main feature of the Balloon model is that it describes the BOLD signal as a nonlinear combination of the blood volume and deoxyhemoglobin concentration, which are expressed as nonlinear functions of the neural activity and the blood flow.

Models in fMRI, in particular the Balloon model, have been used in many studies to estimate underlying parameters. Indeed, these models become criticized because (1) our comprehension of the mechanisms underlying the hemodynamic response is evolving and (2) they are often used without previous evaluation of their identifiability. Therefore, this paper is relevant to this second point. Indeed, estimating the state, the input, and parameters for the Balloon model using BOLD measurements is ill-posed. The ill-posedness, also seen as an identifiability issue when the input signal is given, consists in having a small change in the BOLD signal while observing large variations of parameters and thus poor accuracy of the parameter estimates. Different strategies have been used to add prior knowledge that makes the problem well-posed. Riera et al. [9] used radial basis functions to express the input while Havlicek et al. [10] supposed Gaussian priors for the parameters. Small prior variances are sought to overcome the ill-posedness of some parameters. In the same field of Kalman filtering techniques, Hu et al. [11] used a square root unscented Kalman (SR-UKF) filter to estimate the states and some parameters with Gaussian priors.

Besides, even if it were possible to develop theoretical tools to get an analytical expression for the parameters from the nonlinear equations describing the dynamic behavior of the Balloon model, the input signal has to be “rich enough” so that the parameters can be estimated accurately. The typical example for that is when one parameter quantifies the output behavior with respect to the derivative of the input, and if the input is constant, then the parameter cannot be estimated efficiently. Thus, the input sequence has to be well designed to capture the effect of each of the unknown parameters on the output signal.

In practice, usually there is unfortunately a trade-off between the good contrast-to-noise ratio (due to slow or static behavior) and the estimation efficiency (often assessed for fast time scales). In general, the challenge is attributed to experimental limitations such as possible damage of the studied system (due to high switchings in frequencies, e.g.) and poor signals quality (low signal to noise ratio SNR) when dealing with high frequencies. This question has been raised for the fMRI experimental paradigms by Liu et al. [12] and a theoretical model for the quantification of the so-called detection power/estimation efficiency duality. The approach was developed within the hemodynamic general linear modeling framework.

In this study, we will focus on the identifiability study of the Balloon model parameters which represents an attempt to answer the question:

 
*Given the output measurement y*(*t*)* (BOLD) and the input u*(*t*)* (Neural activity), can θ (Balloon model parameters) be determined uniquely?*

The paper is organized as follows. In the second section, we introduce the nonlinear hemodynamic Balloon model and present the commonly used fMRI paradigms. The third section reviews the existing sensitivity approaches of the Balloon model and completes them by a global sensitivity study when the stimulus follows block design experiment. Finally some conclusions are presented in the fourth section.

## 2. Problem Statement

### 2.1. The Balloon Model

**Figure 1:**
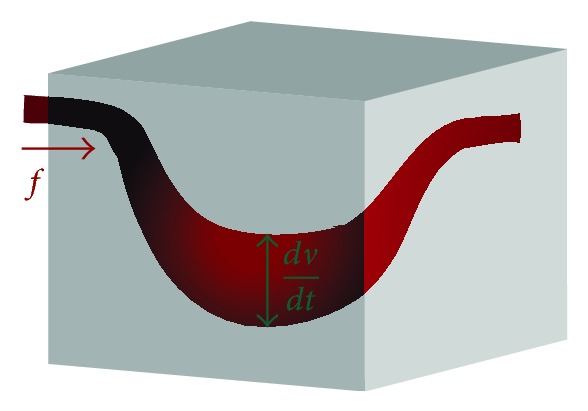
The veinous compartment in a local area of the brain.

The dependence of the BOLD signal upon the variations of the normalized blood flow *f*, volume *v*, and deoxyhemoglobin *q* content has been proposed by Buxton et al. [6] and completed by Mandeville et al. [13]. The model presents the venous compartment's structure as a Balloon where a stimulus resulting in a local neuronal activity leads to an increase in the blood flow as illustrated in Figure 1.

This increase exceeds the cerebral metabolic rate of oxygen thus reducing the concentration of deoxyhemoglobin which in turn results in an increase in the magnetic resonance signal, also called the BOLD effect [14]. Although the proposed model provides a mapping between the BOLD and the physiological variables, there still remains the need to explicit the relationship between the neural activity and the blood flow dynamics. The modeling of this part of the hemodynamic mechanism has been introduced by Friston et al. [8]. Indeed, under the assumption of a linear relation between the synaptic activity and the regional cerebral blood flow, a new variable called the flow-inducing signal has been introduced. The proposed coupling between the neuronal activity *u* and the cerebral blood flow *f* has a linear second order behavior of a relaxed oscillator.

To sum up, the Balloon model can be formulated as a single input/single output nonlinear state-space representation:

(1) x˙tGxt,ut,θ,yt=Hxt,θ,

where the vector *x* = [*f*, *s*, *v*, *q*]^*T*^ is a state vector which consists of a set of the nonmeasurable variables defined above and *s* is called the “flow-inducing signal.” The physical hidden states (*f*, *v*, and *q*) are normalized with respect to the rest phase and thus equal to 1 at rest. *u* is the input of the model which represents the stimulus function, generally associated to the neural activity, and *y* is the output given by the BOLD signal. *G* and *H* are nonlinear evolution and observation functions which are given by

(2) Gxt,ut,θ=s−κff−1−κss+ϵut1τf−v1/α1τf1−1−E01/fE0−v1/α−1q,Hxt,θ=V0k11−q+k21−qv+k31−v.

*θ* represents a set of parameters and consists of the neural efficiency *ϵ*, the flow decay *κ*_*s*_, the flow time constant *κ*_*f*_, the venous transit time *τ*, Grub's stiffness parameter *α*, the oxygen extraction at rest *E*_0_, and the blood volume fraction at rest *V*_0_. These parameters may vary from a brain region to another or from a subject to another. The parameters *k*_1_, *k*_2_, and *k*_3_ depend on the scanner.

The main feature of the Balloon model is that it is nonlinear both in state and parameters. It presents an unusual nonlinearity with respect to the state vector component, for *v* and *q* dynamic equations.

### 2.2. Experimental Design in fMRI

Experiments in fMRI are sought to test biological hypotheses. Nevertheless, there are several experimental constraints related to both physiological effects to which fMRI is sensitive and temporal properties of the BOLD signal itself [15]. The resulting noises include, for example, the cardiac and respiratory cycles as well as motion artifacts. For a more thorough analysis about the nature of fMRI signals' components, readers can refer, for example, to Howseman et al. [16]. These limitations restrict the frequency of stimuli occurrence that provides an unambiguously detectable activation signal. According to the mentioned study, the duration of a cycle ranges from few minutes to few seconds. The cycles consist of successive boxcar functions where the rest periods are intended to provide a constant baseline.

#### 2.2.1. Block Paradigm

In this category [17, 18], the stimulus consists of alternated periods of activation and rest. Each task is of roughly equal duration (the typical block length is 15 s on and 15 s off). In this case, the activation signal and the noise correlation are low and a large BOLD signal change [19] is introduced. The rest periods provide low-frequency drift secondary to physiological noise which can be considered as baseline.

#### 2.2.2. Event-Related Paradigm

This experimental design, requiring a higher temporal resolution, emerged in the mid 90s taking advantage of faster image acquisition techniques. The event durations are much shorter (about one or two seconds) [18] compared to block design paradigm. The main advantages of event-related design include the reduction of the effect of head movements and the ability to capture transients in the BOLD signal [19]. The interstimulus length can be as short as the event duration or may be varied to avoid the subject predict the events and more importantly to improve the detectability of the effects of interest.

#### 2.2.3. Mixed Paradigm

This paradigm results from the combination of block and event-related designs. It consists of close events separated by long rest periods. Mixing transient (trial-related) and sustained (task-related) components in the obtained BOLD signals allows the identification of regions of the brain with mode-related neural activity and others with task-related patterns.

## 3. Identifiability Study of the Balloon Model

Deneux and Faugeras [20] studied the identifiability of the Balloon model parameters at a local level by addressing, in a neighborhood of some nominal values for the parameters *θ*_0_, the invertibility of the mapping:

(3) $$\begin{array}{c} y=F\left(\;u,\theta \;\right), \end{array}$$

where *y* is the measured BOLD signal, *u* is the neural activity, considered as a known input, and *θ* = [*ϵ*, *κ*_*s*_, *κ*_*f*_, *τ*, *α*, *E*_0_]^*T*^ represents the set of unknown parameters.

The rank of the Jacobian of *ℱ* with respect to *θ* determines the number of “unknowns” that can be estimated accurately. These unknowns can match the parameters if the directions defined by the Jacobian are orthogonal; otherwise a small change in one parameter can be partially (if the Jacobian is full rank) or completely offset by a change of a combination of the remaining parameters. Thus to quantify the sensitivity of the output to a small change in a parameter *θ*_*i*_, an orthogonal “projection” of the Jacobian with respect to *θ*_*i*_ on that of *θ*_−*i*_ = *θ*∖{*θ*_*i*_} is considered. In other words, if

(4) Ji∂Fu,θ∂θi,J−i=∂Fu,θ∂θ−i,

then

(5) $$\begin{array}{c} \pi_{i}=\left\|\;\left(\;I-J_{-i}J_{-i}^{+}\;\right)J_{i}\;\right\|=\sqrt{J_{i}^{T}\left(I-J_{-i}J_{-i}^{+}\right)J_{i}}, \end{array}$$

where *J*_−*i*_^+^ is the pseudo inverse of *J*_−*i*_, which is taken as a measure of the sensitivity of *y* to a small change in *θ*_*i*_.

Although this sensitivity study is at a local level and it is predominately oriented to event-related like input *u*, the obtained results for two sets of neural activity signals (a single impulse and two successive impulses) are interesting. They show that *κ*_*s*_ and *κ*_*f*_ estimations are more accurate while *ϵ* seems to be the most difficult to tackle.

More recently, Hu and Shi [21] led to a global sensitivity analysis on some biomedical systems including the Balloon model. This method, developed in a Bayesian framework, consists in assigning a prior distribution to each of the model parameters and then quantifying its contribution to the model output through the study of the posterior distribution. Given the measurement *Y* and an unknown variable *X*_*i*_, then the first order contribution of *X*_*i*_ to the output is quantified as follows:

(6) $$\begin{array}{c} S_{i}=\frac{\text{Var}\left(E\left(Y\;|\;X_{i}\right)\right)}{\text{Var}\left(Y\right)}=\frac{V_{i}}{\text{Var}\left(Y\right)}, \end{array}$$

where *E* is the expectation operator and Var the variance operator. Note that the sum of all *S*_*i*_ is always less or equal to 1; the equality is assessed in the case of additive models. Higher order contributions represent joint variance terms with the remaining variables. For example, second order contributions, also called second order sensitivity indexes are terms of the form:

(7) $$\begin{array}{c} S_{ij}=\frac{\text{Var}\left(E\left(Y\;|\;X_{i},X_{j}\right)\right)-V_{i}-V_{j}}{\text{Var}\left(Y\right)}, \end{array}$$

and so forth. According to this criterion the higher the index is the more likely the variable to drive the model output variance is, and thus the better the candidate for model calibration is. Similarly, variables with small indexes can, for example, be frozen to their nominal values. In practice, this criterion requires evaluation of probability densities for nonlinear (nonadditive) quantities which makes this technique computationally costly to implement. Two approximations based on Monte Carlo method and the Unscented Transformation were used.

Sensitivity analysis has been applied to the Balloon model in the case of event-related and block designs. The main outcomes of this study show that *V*_0_ (the Balloon model parameter, not to be confused with the above notations for variances) has the highest importance in driving the BOLD uncertainty, whereas *α* seems to have a minor effect making it a noninfluential parameter for the BOLD signal. Regarding the dynamics of the BOLD signal, *E*_0_ seems to affect more the initial dip and the rising to peak phase while *κ*_*s*_ contributes more at the undershoot level.

In order to come up with a conclusion on the identifiability of the Balloon model and to consolidate the previous results for the special case of block design experiments, the next part of this work will be dedicated to a sensitivity study.

### 3.1. Variation of the BOLD Signal When Varying One Parameter

This is an intuitive approach which consists in tracking the changes in the output signal, that is, the BOLD when only one of the model's parameters varies. It allows seeing to which parameters the BOLD is the more sensitive. For simplicity, we will name *θ*_*i*_ the Balloon model parameters in (2) with a change to the inverse for *α* and *τ*. This parametrization together with the nominal values taken for the reference model (as in Friston et al. 2000 [8]) are summarized in Table 1.

As for the remaining observation equation parameters, it is agreed that they are scanner dependent [6]. The commonly used values, corresponding to 1.5*T*, are taken as in the literature:

(8) k17E0,k2=2,k3=2E0−0.2.

*Remark*. *k*_1_ and *k*_3_ are dependent on *E*_0_, so they represent additional unknowns to the estimation problem.

**Figure 2:**
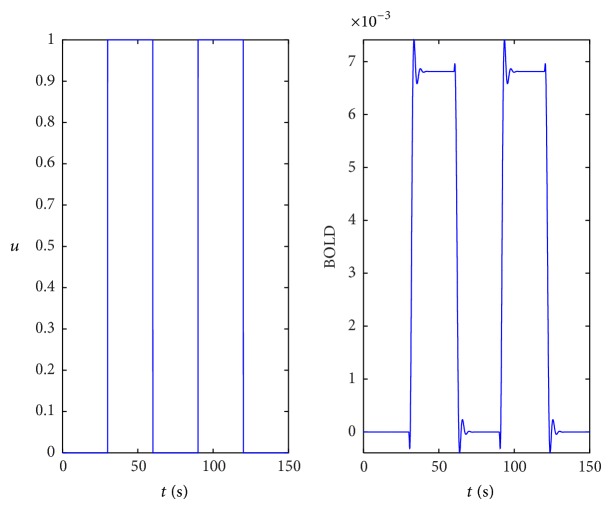
Input and BOLD signals.

The input signal consists of a square function with an amplitude of one and a period of 1 minute (30 seconds OFF and 30 seconds ON). The sampling period *T*_*e*_ is equal to 0.01 s. The input *u* and the BOLD signal are shown in Figure 2. The experiment corresponds to short transients compared to the overall stimulus duration and the BOLD signal presents static regime intervals which is a typical feature of block designs.

**Figure 3:**
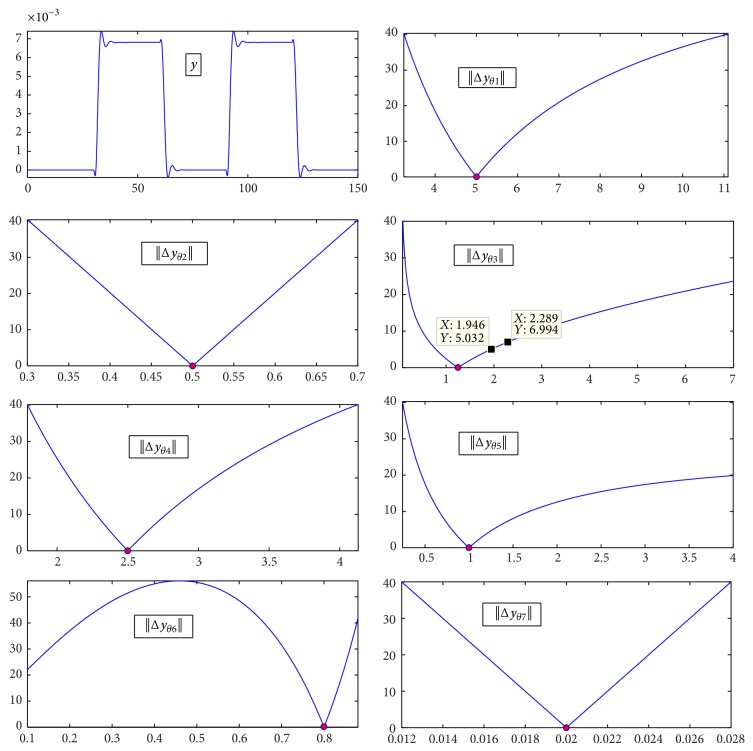
Norm of the BOLD relative error in percent (100 · ‖Δ*y*‖/‖*y*‖) as a function of variation in parameters; the error value is limited to 40% for most of the parameters. It goes to more than 50% for Θ_6_ to view the maximum.

Each subplot of Figure 3 (except the first one which is the nominal BOLD) corresponds to the relative error in percent of the BOLD signal when one of the parameters takes values in a grid around the nominal value (shown in Table 1).

*The Minimum*. All the plots, except that of *E*_0_, present a unique extreme in a neighborhood of the nominal value of the parameter corresponding to the global minimum. Subsequently, the estimation of *E*_0_ can be more complex, requiring a good minimum search method and an adequate initial guess. 

*Symmetry of the Criterion*. It is also worth noticing that the criterion is not symmetric for *α*, *κ*_*s*_, *κ*_*f*_, and *τ*. Indeed, the greater the value of the parameter is, the smaller its contribution to the criterion is. This makes it obvious that, to estimate these parameters efficiently, initial guesses should be taken less than the expected value for each of the parameters.

The dissymmetry of the cost function might be explained as follows: these parameters monitor the transients of the BOLD signal in the sense that small values imply slow dynamics for the Balloon model. A slow system is unable to track changes of the input which results in a big disparity between the nominal and actual BOLD signals. Similarly, a faster dynamic system reaches the steady state more quickly than the nominal system. The disparity only applies to the transients, which are very short compared to steady state regimes.

*Sensitivity of the BOLD*. The shape of the criterion suggests that the BOLD is less sensitive to changes in the abovementioned parameters when their values exceed the nominal values. As an illustration, a variation of more than 50% of *κ*_*s*_ is needed to increase the relative error of the BOLD signal by 5%. While the latter value of the error is a very satisfying rate when fitting the BOLD signal, it is highly inaccurate to estimate a parameter with 50% uncertainty.

Apart from the contribution of each parameter to the BOLD, it is also interesting to check if there is a correlation between these single contributions. In this case, the Balloon model, subject to blocked paradigm, is defined by the different correlation directions rather than the parameters.

To “partially” answer this question, the contributions of parameters, two by two, to the BOLD signal are explored in the next subsection.

### 3.2. Sensitivity of the BOLD to Simultaneous Changes in Two Parameters

The approach adopted in this part of the study consists in creating a grid for each of the parameters. Each plot corresponds to the relative error of the BOLD, when a pair of parameters varies on the grid and the remaining parameters are maintained to their nominal values. The grids are chosen such that the relative error on the BOLD does not exceed 30%, assuming that any efficient approach for the estimation of the model parameters will not provide a worse result for the BOLD fitting.

**Figure 4:**
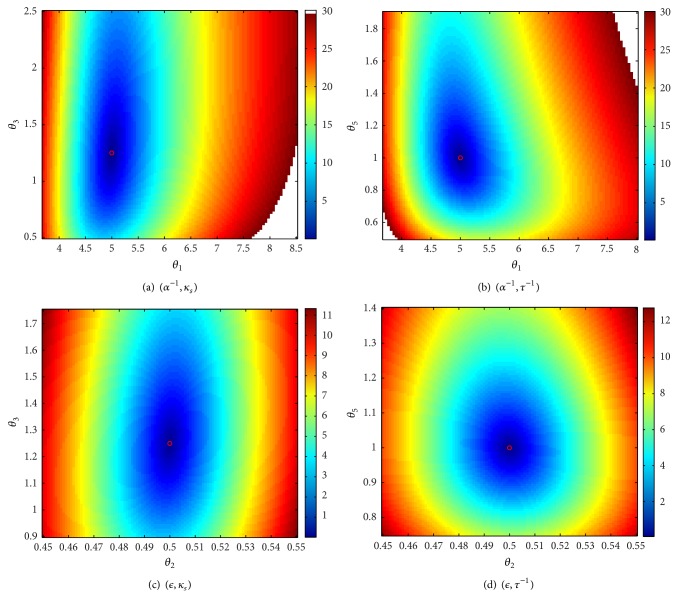
Uncorrelated contributions (1/2).

**Figure 5:**
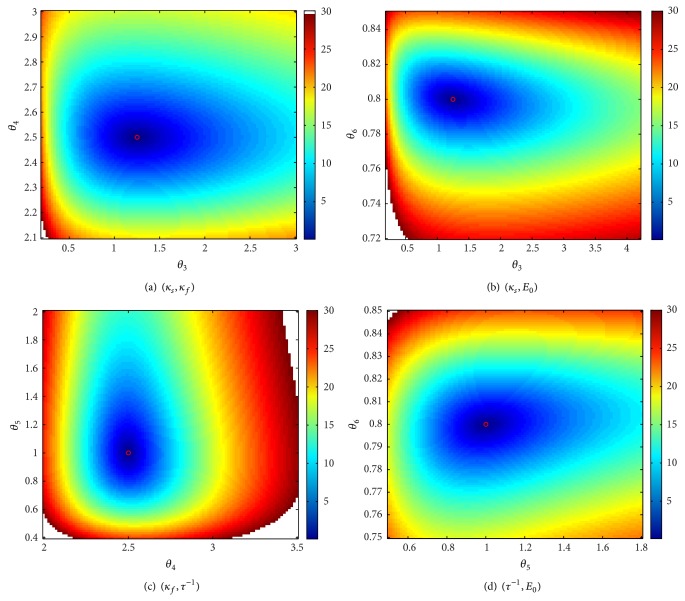
Uncorrelated contributions (2/2).

The pairs having less correlated contributions are summarized in Figures 4 and 5. It is interesting to notice that the lowest correlation is obtained for the neural efficiency *ϵ* (*θ*_2_) and the venous transit time *τ* (*θ*_5_) as shown in Figure 5(b). More generally, the venous transit time seems to define a direction that is independent from contributions of most of the Balloon parameters (more precisely, it only slightly correlates with *κ*_*s*_).

If the global minimum is well marked for small variations around the nominal values (corresponding to a relative error less than 5%), correlation directions are more and more clearly defined as the relative error increases. These correlations are almost exclusively located at regions corresponding to parameter values which are greater than the nominal value, which is typically the case for *θ*_3_ (*κ*_*s*_) and *θ*_5_ (*τ* ^−1^). This further confirms that the BOLD signal is less sensitive to large increase in the parameters defining the dynamics of the Balloon model.

**Figure 6:**
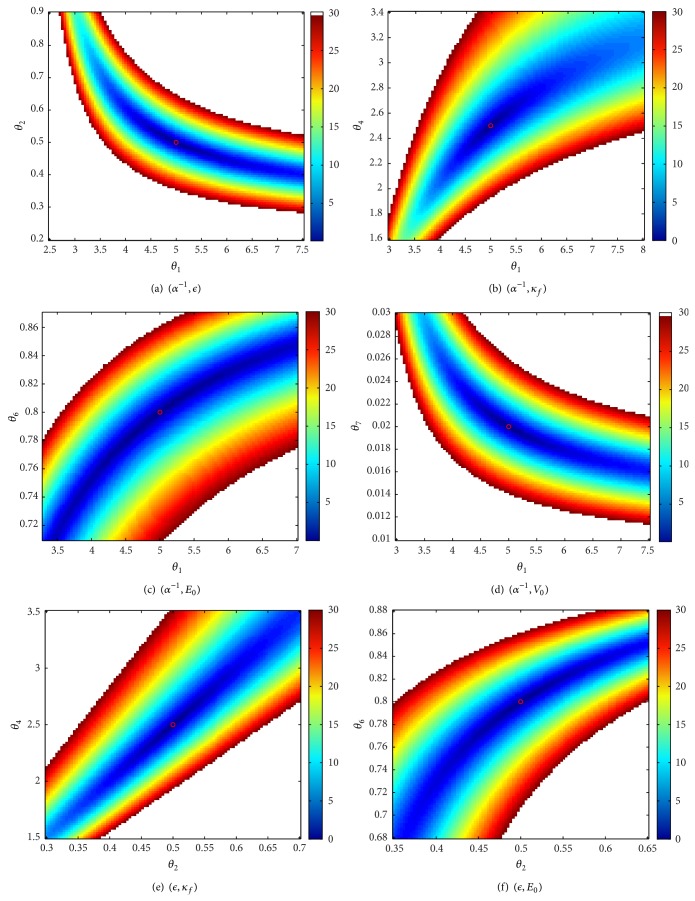
Correlated directions (1/3).

**Figure 7:**
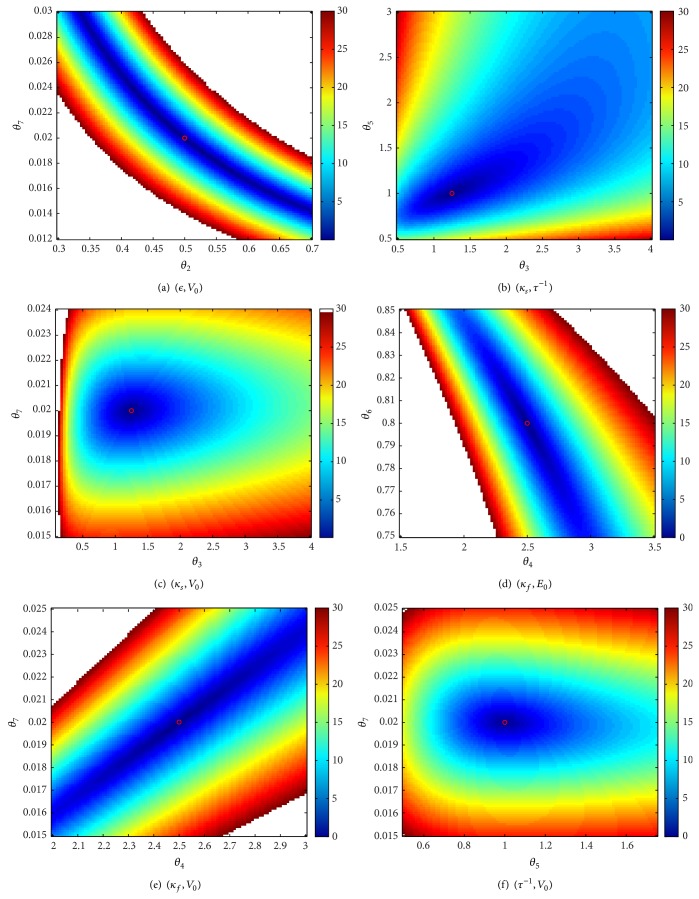
Correlated directions (2/3).

**Figure 8:**
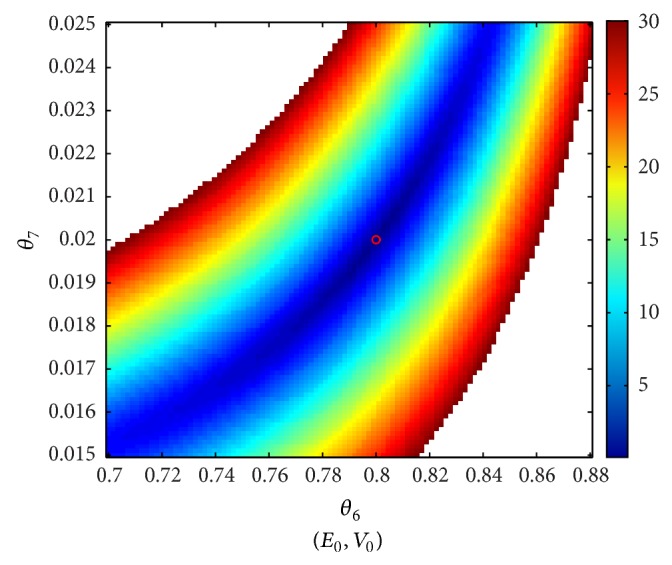
Correlated directions (3/3).

The second series of plots, shown in Figures 6 to 8, illustrates cases where the global minimum is not “sufficiently marked” even in a small neighborhood of the nominal values of parameters. Clearly, the estimation of *V*_0_ is problematic. Indeed, whatever parameter contribution, taken in a reasonable neighborhood of the nominal value, can be offset by *V*_0_.

## 4. Conclusion

The sensitivity study of the Balloon model highlights the importance that should be given to the design of the experimental paradigm. This work completes the previous studies, showing the difficulty to estimate accurately the Balloon model parameters using the well-known blocked design. The lack of accuracy is due in part to the trade-off between detectability and estimation when it comes to fMRI experiments. Indeed, increasing the randomness of the input signal, as usually required to cover as much frequencies as possible, makes it difficult to have a contrast signal which is high enough to detect the active areas of the brain.

The need to develop new paradigms that could mix up randomness and longer events seems obvious especially in the absence of a well-established theoretical framework that can handle the exact identifiability study of the Balloon model. The calibration could also be assessed through several experiments, event-related like inputs, for example, allow the estimation of dynamic parameters (while blocked designs allow the detection of evoked activity more reliably). The estimation of *V*_0_, however, remains an issue as it can easily introduce a significant bias on the overall estimates.

More generally speaking, this study goes beyond the Balloon model itself to further confirm the difficulty of characterizing the pathway from the stimulus to the BOLD effect. Several open questions, tightly connected to the inability of the BOLD signal to calibrate the hemodynamic model accurately, can be raised. Possible solutions include revising the hemodynamic modeling, or combining fMRI with other imaging techniques to have extra measurements, for example, the cerebral blood flow.

## Figures and Tables

**Table 1:** Parameters of the Balloon model.

Variable	Parameter	Value
θ_1_	α^−1^	5
θ_2_	ϵ	0.5
θ_3_	κ_*s*_	1.25
θ_4_	κ_*f*_	2.5
θ_5_	τ^−1^	1
θ_6_	*E*_0_	0.8
θ_7_	*V*_0_	0.02
